# RapidAIM 2.0: a high-throughput assay to study functional response of human gut microbiome to xenobiotics

**DOI:** 10.20517/mrr.2023.57

**Published:** 2024-04-03

**Authors:** Leyuan Li, Janice Mayne, Adrian Beltran, Xu Zhang, Zhibin Ning, Daniel Figeys

**Affiliations:** School of Pharmaceutical Sciences, Ottawa Institute of Systems Biology and Department of Biochemistry, Microbiology and Immunology, Faculty of Medicine, University of Ottawa, Ottawa K1H8M5, Ontario, Canada.; ^#^Authors contributed equally.

**Keywords:** Gut microbiome, metaproteomics, high-throughput *in vitro* assay, biobanking, functional responses

## Abstract

**Aim:** Our gut microbiome has its own functionalities which can be modulated by various xenobiotic and biotic components. The development and application of a high-throughput functional screening approach of individual gut microbiomes accelerates drug discovery and our understanding of microbiome-drug interactions. We previously developed the rapid assay of individual microbiome (RapidAIM), which combined an optimized culturing model with metaproteomics to study gut microbiome responses to xenobiotics. In this study, we aim to incorporate automation and multiplexing techniques into RapidAIM to develop a high-throughput protocol.

**Methods:** To develop a 2.0 version of RapidAIM, we automated the protein analysis protocol, and introduced a tandem mass tag (TMT) multiplexing technique. To demonstrate the typical outcome of the protocol, we used RapidAIM 2.0 to evaluate the effect of prebiotic kestose on *ex vivo* individual human gut microbiomes biobanked with five different workflows.

**Results:** We describe the protocol of RapidAIM 2.0 with extensive details on stool sample collection, biobanking, *in vitro* culturing and stimulation, sample processing, metaproteomics measurement, and data analysis. The analysis depth of 5,014 ± 142 protein groups per multiplexed sample was achieved. A test on five biobanking methods using RapidAIM 2.0 showed the minimal effect of sample processing on live microbiota functional responses to kestose.

**Conclusions:** Depth and reproducibility of RapidAIM 2.0 are comparable to previous manual label-free metaproteomic analyses. In the meantime, the protocol realizes culturing and sample preparation of 320 samples in six days, opening the door to extensively understanding the effects of xenobiotic and biotic factors on our internal ecology.

## INTRODUCTION

Numerous studies have shown that xenobiotic compounds can functionally affect the gut microbiota. These include pharmaceutical compounds, not only those developed for combating microbial infections, but also those non-antimicrobial drugs developed to target the host functions^[1]^. Biotic factors from external sources, such as probiotics, pathogens, phages, *etc*., also influence the gut microbiome functionality in various ways. To deconvolute the complexity of microbiome responses to these factors, *in vitro* approaches in the absence of the host component have been used. Studies have shown that commensal bacterial species in the gut can be directly affected by 24% of commonly used host-targeted drugs^[1]^, and therapeutic drugs can accumulate in gut bacteria without altering their abundances^[2]^. However, studying gut microbes in isolation has its limitations, because microbial species function differently in complex community settings in comparison to pure cultures. Cooperative and antagonistic interactions collectively contribute to microbiome diversity and resilience^[3]^. Several synthetic communities have also been used to evaluate the effect of drugs on community composition and cross-feeding interactions^[2]^. Nevertheless, the natural human gut microbiome harbors hundreds of microbial species, with a considerable variation in taxonomic functions and compositions among different individuals^[4-6]^. The complexity and variability are much greater than those of synthetic gut microbial communities. Studies have shown long-term stability of the individual gut microbiome across the life span^[7]^, and this stability can be irreversibly perturbed by xenobiotic stimulation^[8,9]^. Therefore, when it comes to the context of individual gut microbiomes, it is necessary to adopt an assay that maintains the individuality of the community composition and function *in vitro*. In addition, since evaluating xenobiotic compounds or biotic components against different individual microbiomes requires large matrices of samples, high-throughput-compatible models and assay readouts are necessary to perform such studies. There have been various models to evaluate microbiome responses. Early *in vitro* gut microbiome models were based on large-scale bioreactors that are low-throughput and, due to the large volume of culturing, very costly owing to the considerable amounts of compounds added. More recent advances in modeling the gut ecosystem include realizing the culturing of complex human gut microbiome in anaerobic intestine-on-a-chip models, enabling the observation of host-microbiome interactions^[10]^. However, for the purpose of high-throughput compound screening, these models are not easily adaptable. The use of deep-well plates has the advantage of easy setup,cost-effectiveness, and time efficiency when scaling up; additionally, it is compatible with automated downstream analysis.

In terms of assessing the functional response of cultured microbiomes, liquid chromatography - tandem mass spectrometry (LC-MS/MS) is capable of metaproteomic analysis of microbial communities^[11]^. Briefly, LC-MS/MS separates peptides by mass-to-charge ratio, and fragments separated peptides to generate MS/MS spectra, which are subsequently matched to peptide sequences through database search approaches. With its fast-growing measurement depth and capacity, metaproteomics techniques have been used to study microbiome-associated health and diseases such as inflammatory bowel disease^[12,13]^, colorectal cancer^[14]^, diabetes^[15]^, mental illnesses^[16]^, and COVID-19^[17]^. It has also been used to evaluate *in vitro* responses of gut microbiomes to various xenobiotics. We have developed RapidAIM, namely Rapid Assay of Individual Microbiome, taking advantage of fast-pass metaproteomics to study the human gut microbiome’s protein expression responses to xenobiotics in a high-throughput compatible setting^[18,19]^. RapidAIM has been widely used in numerous research studies. As a proof of concept of RapidAIM, we first used it to assess the effect of 43 xenobiotic compounds on five individual gut microbiomes and discovered that seven of the tested compounds showed consistent effects across individual samples, while some other compounds showed significant but individually distinct effects^[18]^. RapidAIM was then used to evaluate the effect of a panel of structurally similar compounds of berberine^[20]^, different structures of resistant starches^[21]^, and commonly used sweeteners^[22]^ on individual gut microbiomes. RapidAIM was also used to evaluate the effect of a bacteriophage preparation on microbiome composition and function^[23]^, as well as being applied in two ongoing clinical trials for the selection of therapeutic interventions (NCT04520594 and NCT04522271). The optimized culture model (MiPro) of RapidAIM has been used by other researchers to investigate gut microbiome responses to oligomannate^[24]^, dietary cholesterol^[25]^, dietary fibers^[26]^, antibiotics^[27]^, and nanoparticles^[28]^. Notably, the previous efforts of RapidAIM did not realize automated high-throughput metaproteomic analysis. With the rapid rise of the automation and big data era, the development of a 2.0 version of the protocol to expand the capability to study microbiome responses to various stimuli is timely, and its broad application is highly expected.

In RapidAIM 2.0, we incorporate automation and multiplexing techniques into the metaproteomic analysis workflow to observe the response of protein expression in the *in vitro* microbiome in a high-throughput manner. The use of isobaric chemical labels, such as tandem mass tag (TMT) approach, offers great potential in the analysis of large sample sets such as those generated from our individual microbiome-xenobiotics assay designs. TMT-based quantitation has recently been used for large-scale proteomics studies owing to its high multiplexing capacity and deep proteome coverage^[29]^. The use of TMT labeling also significantly reduces LC-MS/MS time and cost^[30]^. A recent study reported the development of a high-throughput stool metaproteomics workflow^[31]^. The protocol used Protifi S-trap to clean up the proteins in combination with TMT labeling and automation, which also greatly saves time. However, only around 5,000 microbial proteins were identified from a total of 290 human stool samples^[31]^. Our recently optimized TMT-metaproteomics workflow identified 28,605 microbial peptides and 10,656 microbial protein groups from 97 samples. This streamlined TMT labeling workflow is fully compatible with automation of the metaproteomics sample preparation steps which altogether can significantly increase the robustness of liquid handling compared to manual operation, speed up the experimental workflow, and further increase the throughput^[32]^. In this paper, we further adapted this workflow to an automated version, and show that metaproteomic profiling derived from cultured samples of four human gut microbiomes resulted in a total of over 5,000 quantified protein groups per sample. This is comparable to previous studies using manual, label-free metaproteomics protocols^[18,33]^. To demonstrate the typical outcome of the protocol, we show an example of using RapidAIM 2.0 to evaluate the effect of prebiotic kestose on *ex vivo* individual human gut microbiomes preprocessed with five different workflows; we also show that kestose had consistent functional effects across individuals and can be used as a positive control in the assay.

## METHODS

### Proof-of-concept of the RapidAIM 2.0 approach

We designed a study to exemplify the expected outcome of the RapidAIM 2.0 approach. The study includes an evaluation of different sample biobanking methods and sample storage methods. Although these tests do not provide specific examples of different compounds, the comparison of the sample pre-processing workflow described above still yields a diverse range of samples. This result serves to effectively showcase the stability and versatility of the high-throughput strategy employed. To evaluate sample biobanking methods, four different stool sample processing strategies, namely gauze filtration (Gauze), 100 µm vacuum filtration (Vaccum), 100 µm spin tube filtration (Spin), and 100 *g* spinning (100 *g*) workflows, were tested to evaluate whether any of the methods may have an impact on the initial microbiome, cultured microbiome and microbiome responses. A commercial kit GutAlive® was evaluated following the manufacturer’s manual for its performance on live microbiome sample collection (0 day) and preservation at room temperature for 24 and 72 h before being processed using the 100 µm vacuum filtration protocol. We also examined the storing efficacy of our in-house PBS-glycerol buffer by comparing samples processed immediately after collection (0 day) with those stored for 24 and 72 h at 4 °C.

### Reagents and stock solutions

(1) Reagents and consumables: reagents and consumables for gut microbiome culturing, metaproteomic sample processing, and TMT labeling are listed in detail in Supplementary Information 1.

### Culture medium stock solutions

(2) Culture medium stock solutions: stock solutions of the culture medium were prepared following details described in Supplementary Table 1.

(3) 1M Tris-HCl stock solution, pH = 8.0: 12.11 g of Tris base was weighed and added to 80 mL of ddH_2_O. While being mixed on a magnetic stirrer, pH was adjusted to 8.0 using HCl. Top up the solution to 100 mL using ddH_2_O and double check the pH.

(4) TMT aliquot stock plates^[34]^: the TMT11plex^TM^ reagents (5 mg per channel) were equilibrated to room temperature, then 300 μL of anhydrous acetonitrile (ACN) was added to each tube of the reagents and the mixture was allowed to dissolve for 5 min with occasional vortexing. Each of the reagents was transferred to 15 mL Falcon tubes, and 4,700 μL anhydrous ACN was added to each of the 15 mL tubes. Mix thoroughly. Aliquot the TMT reagents to 96 well plates (50 μL per well); each 11plex should be arranged in order in a row. 4 ^o^C Freeze-dried stock plates were stored at -80 ^o^C.

(5) Microbial cell lysis buffer: cell lysis buffer contained 8 M urea and 4% sodium dodecyl sulfate (SDS) in 100 mM Tris-HCl (pH = 8.0). For every 50 mL lysis buffer, one Roche cOmplete^TM^ tablet was added and sonicated to dissolve. Or use a cOmplete^TM^ mini tablet for every 10 mL of lysis buffer. Lysis buffer must be freshly prepared.

(6) Protein precipitation solution: precipitation solution of 50%:50%:0.1% (v/v/v) of acetone: ethanol:acetic acid solution was prepared and stored at -20 °C for at least overnight before use.

(7) Protein resuspension buffer: 6 M urea in 100 mM Tris-HCl (pH 8).

(8) 0.1 M dithiothreitol (DTT) solution: 77 mg of DTT powder weighed into 5 mL ddH_2_O was prepared freshly before use (or prepare in advance and store solution at -80 ^o^C).

(9) 0.2 M iodoacetamide (IAA) solution: 185 mg of IAA powder weighed into 5 mL ddH_2_O was prepared freshly before use (or prepare in advance and store solution at -80 ^o^C).

(10) Trypsin solution: 1 mL of 100 mM Tris-HCl buffer containing 2 μg/mL trypsin was prepared for each sample plate.

(11) Desalting buffers: Wash buffer [0.1% (v/v) formic acid (FA) in water], elution buffer [0.1% FA in 80% ACN: 80% (v/v) ACN and 0.1% (v/v) FA in water], acidifying buffer [10% FA: 10% (v/v) FA in water] and 5% (v/v) FA in water was prepared for the desalting procedure.

(12) TMT labeling solutions: 100 mM tetraethylammonium bromide (TEAB) in 20% ACN: 10% (v/v) 1M TEAB, 20% (v/v) 100% ACN and 70% (v/v) HPLC-grade water was mixed to prepare the TEAB solution.

0.8% hydroxylamine in 100 mM TEAB (quencher): 10% (v/v) 1M TEAB and 90% (v/v) HPLC-grade water were mixed to prepare 100 mM TEAB water solution. Then, 1.6% (v/v) 50% hydroxylamine was mixed with 98.4% (v/v) 100 mM TEAB water solution to prepare the quencher.

### Medium preparation

Culture media was consisted of the following composition^[19]^: 2.0 g/L peptone water, 2.0 g/L yeast extract, 0.5 g/L L-cysteine hydrochloride, 2 mL/L Tween 80, 5 mg/L hemin, 10 μL/L vitamin K1, 1.0 g/L NaCl, 0.4 g/L K_2_HPO_4_, 0.4 g/L KH_2_PO_4_, 0.1 g/L MgSO_4_·7H_2_O, 0.1 g/L CaCl_2_·2H_2_O, 4.0 g/L NaHCO_3_, 4.0 g/L porcine gastric mucin, 0.25 g/L sodium cholate, and 0.25 g/L sodium chenodeoxycholate. Follow detailed procedures in Supplementary Table 2 for medium preparation.

### Automated digestion deck set up

A deck layout of the Hamilton Nimbus 96 automation system containing five plate/reservoir plate locations, two tip racks, a thermo block, and a small-volume reservoir location (for DTT and IAA) was set up for automated digestion. The plate locations contained four sample plates and one reservoir plate for trypsin-Tris-HCl solution. Two tip racks held tips to be used in adding DTT and IAA, and trypsin-Tris-HCl solution, respectively.

### Automated desalting deck set up

A deck layout containing seven plate/reservoir locations and two tip racks was set up for automated desalting. The plate locations contained one sample plate, one elution plate, two sample washing plates, and three reservoir plates for 100% ACN, 0.1% FA and 80% ACN + 0.1% FA solutions, respectively. The tip racks held pipette tips and reverse-phase (RP) desalting columns, respectively.

### Collecting and processing stool samples

An anaerobic chamber was used for sample processing and culturing. The anaerobic chamber contained 5% H_2_, 5% CO_2_ and balanced with N_2_. We used a palladium catalyst to react O_2_, and Thermo Scientific^TM^ Oxoid^TM^ Resazurin Anaerobic Indicator to make sure the chambers remain anaerobic. We used an in-house prepared buffer to collect fecal samples. Fecal samples were prepared to a 20% (w/v) slurry in sterile, pre-reduced 1X PBS (pH 7.4) containing 10% (v/v) glycerol and 1 mg/mL L-Cysteine. In addition, the commercial GutAlive kit was compared for its performance in preserving live microbiota at room temperature following the manufacturer’s instruction. The stool samples were processed through four different approaches, i.e., gauze filtration, 100 µm vacuum filtration, 100 µm spin tube filtration, and 100 *g* spin. Detailed protocols for collecting and processing stool samples, as well as processing of stool samples for long-term live microbiota biobanking^[35]^, are presented in Supplementary Methods 1 and 2. Note that institutional ethical approval must be obtained, ensuring all samples are collected with informed written consent and in accordance with relevant guidelines. In this study, the protocol for human stool sample collection (# 20160585-01 H) was approved by the Ottawa Health Science Network Research Ethics Board at the Ottawa Hospital, Ottawa, Canada. Four healthy individual’s microbiomes (V54, V55, V56 and V57, with ages of 53, 30, 33, and 50 years old) were included. The inclusion criteria were healthy individuals who are 18-65 years of age. Exclusion criteria for participation included the presence of irritable bowel syndrome, inflammatory bowel disease, or diabetes diagnosis; antibiotic use or gastroenteritis episode in three months preceding collection; use of pro-/pre-biotic, laxative, or anti-diarrheal drugs in the last month preceding collection; or pregnancy^[35]^. Written consents were obtained from all participants.

### Microbiome culturing and treatment

In the anaerobic chamber, 1 mL culture media and 100 µL stool samples were added into each well of a square-well 96-deepwell plate using a 96-channel liquid handler (e.g., epMotion® 96) (or can use a multi-channel pipette). 2 mg/mL kestose was added into the culture media for the kestose treatment groups. Samples were mixed sufficiently before covering the wells tightly with a perforated silicone gel mat and sealed around using lab tape to prevent popping up due to gas production in the culture. The deepwell plates were shaken at 500 rpm on an orbital shaker for 18 h at 37 °C.

### Microbial cell washing

After culturing, culture plates were centrifuged at 3,000 *g* for 45 min at 4 °C. The microbial cell-free supernatant was then removed (the supernatant can be collected for pH, metabolome and/or exosome analyses). The pellets were resuspended in 1 mL cold PBS buffer using a 96-channel liquid handler (or a multi-channel pipette). If samples cannot be sufficiently resuspended using pipettes, firmly cover the plate with a silicon gel mat and vortex the plate at 2,000 rpm to mix well. The plates were then centrifuged again at 3,000 *g*, 45 min, 4 °C. After adding 1 mL cold PBS buffer to resuspend the pellet again, the plates were centrifuged at 300 *g*, 5 min, 4 °C to pellet debris. The supernatant was next carefully transferred into a new deepwell plate. The above cell washing steps were repeated for another two rounds, before removing the supernatant and storing the plates at -80 °C before cell lysis.

### Microbial cell lysis and protein double-precipitation

150 μL lysis buffer was added to each of the wells containing microbial cell pellets using a 96-channel liquid handler. The pellets were resuspended before being transferred to a 96-well PCR plate. The PCR plate was covered with strip lids and subjected to sonication with a cup-horn ultra-sonicator (QSonica, cat. no. Q700MPXC) at 10 kHz, 10s-on and 10s-off cycle for 20 min (i.e., total sonication of 10 min), with a recirculating chiller set to 8 °C to prevent overheat of samples. After sonication, transfer the cell lysate into a 1.2 mL 96-well cluster tube plate, and add 800 μL ice-cold protein precipitation solution. Cover with cluster lids and mix well.

Samples were precipitated overnight at -20 °C. After the first precipitation, the sample plates were centrifuged at 3,000 *g*, 45 min, 4 °C. After carefully removing the supernatant, 150 μL protein resuspension buffer was added to the pellets before being mixed at 2,000 rpm using a vortex mixer, until protein pellets in all wells were fully suspended. Next, 800 μL ice-cold protein precipitation solution was added for a second precipitation at -20 °C overnight.

### Protein digestion

The sample plates were centrifuged at 3,000 *g*, 45 min, 4 °C and the supernatant in each well was carefully removed. 100 μL protein resuspension buffer was added to each well and mixed at 2,000 rpm using a vortex mixer, until protein pellets in all wells were fully suspended. Protein concentrations were determined using the DC Protein Assay kit following the manufacturer’s instructions. Protein samples were next diluted to reach a concentration of 1 μg/μL using the protein resuspension buffer.

Protein digestion was then performed using an automated liquid handler (Hamilton Nimbus 96, cat. no. OPP041219): first, 10 μL 0.1 M DTT solution was added to each well of 100 μL samples, and incubated at 56 °C, 800 rpm for 30 min. Next, after cooling the plates to room temperature, 10 μL 0.2 M IAA solution was added to each well and was incubated at room temperature in the dark for 40 min. Finally, 1,000 μL 100 mM TrisHCl buffer containing freshly prepared 2 μg/mL trypsin (trypsin:proteins = 1:50) was added into each well before being incubated at 37 °C, 800 rpm overnight in ThermoMixers.

### Desalting

After digestion, desalting of protein lysate was performed using an automated liquid handler (Hamilton Nimbus 96): first, each sample was acidified with 100 μL 10% FA to reach a pH of 2-3. The reverse-phase (RP) desalting columns (e.g., IMCS, 04T-H6R05-1-10-96, 04T-H6R52-1-10-8 or equivalent) was conditioned by two cycles of up-and-down mixing in 100% ACN and two cycles of mixing in 0.1% FA. The recommended volume of mixing is 500-800 μL in each cycle. Next, samples were loaded to the pre-activated reverse-phase (RP) columns by at least ten cycles of up-and-down mixing of 500-800 μL volume. The RP columns were then washed by two cycles of up-and-down mixing in a first sample washing plate containing 0.1% FA, followed by being mixed in a second sample washing plate of 0.1% FA for another two cycles.

800 μL 80% ACN + 0.1% FA was transferred from the reservoir plate to the elution plate, and samples in the RP columns were eluted by two cycles of 800 μL up-and-down mixing in the elution plate. From the elution plate, 240 μL of the eluted solution was aliquoted to another 96-well plate to be used for TMT labeling. A mixture of all sample aliquots is recommended to be used as the reference sample for the TMT labeling. Samples were dried in a SpeedVac with a plate adapter at room temperature (check every 20 min until samples are dried).

### TMT-labeling and desalting

20 μL 100 mM TEAB in 20% ACN solution was added to each sample well and mixed sufficiently using 600 rpm on an orbital shaker. 15 μL mixture was aliquoted from each sample well to the corresponding wells of the TMT reagent plates. The plate was covered with plate lids and incubated in the thermomixers at 25 °C, 600 rpm for 2 h. Then, 15 μL quencher (0.8% hydroxylamine in 100 mM TEAB) was added to each well and reacted in the thermomixers at 25 °C, 600 rpm for 15 min. Next, the samples were acidified by adding 60 μL 5% FA to each well, followed by combining each set of TMT11plex^TM^ by taking 80 μL from each sample. Finally, all samples of a same row were combined into a 96-deepwell plate. Samples were desalted and dried in a SpeedVac at room temperature.

### LC-MS/MS analysis

TMT quantitation was performed using a high-resolution LC-MS/MS. Here, we used an UltiMate 3000 RSLCnano system coupled with an Orbitrap Exploris 480 mass spectrometer system; their setups are as shown in Supplementary Tables 3 and 4, respectively. Samples were resuspended at 1 μg/μL protein in 0.1% FA. After being sufficiently mixed using a vortex mixer, samples were centrifuged at 14,000 *g* for 5 min before being loaded to a LC-MS/MS sampler plate. 1-2 μL of each sample was injected to the LC-MS/MS and was analyzed following a 2-hours gradient.

### Database search and data analysis

A database search of the LC-MS/MS raw files was performed using MetaLab 2.3. The software can be freely downloaded at http://imetalab.ca. Here we used MaxQuant for a closed database search. The IGC database was used as the microbiome protein FASTA database. Under the “Parameters” tab, “Carbamidomethyl (C)” as fixed modifications, and “Acetyl (Protein N-term)” and “Oxidation (M)” as variable modifications were selected. “Isobaric labeling” quantification mode of TMT11plex^TM^ was selected. We used default MaxQuant search parameters pre-defined in MetaLab. Data pre-processing was performed using the MSstatsTMT R package based on proteinGroups.txt, evidence.txt tables, and a user-customized msstatstmt_annotation file as the inputs. The processed data table can then be used for downstream data analyses of principal component analysis and hierarchical clustering using RMarkdown.

Timing of each step: Microbiome culturing and treatment, 1-2 days; Microbial cell washing, 5-6 h; Microbial cell lysis and protein double-precipitation, 2 days; Protein digestion, 1 day; Desalting, 1-2 h; TMT-labeling and desalting, 4-5 h; LC-MS/MS analysis, 2.5 h per TMT11plex.

## RESULTS

### Development of the RapidAIM 2.0 approach

The RapidAIM 2.0 approach is an updated version of the previous RapidAIM approaches developed and used in research studies by our laboratory^[18,35]^. In the previous workflows, we described a 96-well-based workflow to study the functional responses of individual gut microbiomes to xenobiotics *in vitro*. The development of the previous RapidAIM method consisted of three major stages, (1) optimized the culture medium and established and validated the 96-well-based scalable culturing model^[19,36]^; (2) established a 96-well-based metaproteomic sample processing and data analysis workflow^[18]^; (3) and most recently, we developed and validated a live microbiota biobanking workflow that is helpful to increase the reproducibility of experiments^[35]^. A limitation of this previous protocol was the considerably large sample size and LC-MS/MS time consumption for metaproteomics analysis. Therefore, we updated the protocol to overcome this limitation. New features of the protocol include (1) optimization of the protein extraction and purification protocol; (2) automation of the protein digestion and desalting protocol; (3) introduction of TMT multiplexing technique for labeling and quantitation of peptides and proteins, allowing for the analysis of up to 10 samples in one LC-MS/MS run^[34]^; and (4) a TMT-based statistical analysis streamline for clustering functional responses. We estimate that, for an experiment containing 320 samples (four 96-well plates), this updated workflow requires only six days for culturing and sample processing, and it shortens LC-MS/MS sample analysis time from approximately 20 to 3 days.

**Figure 1:**
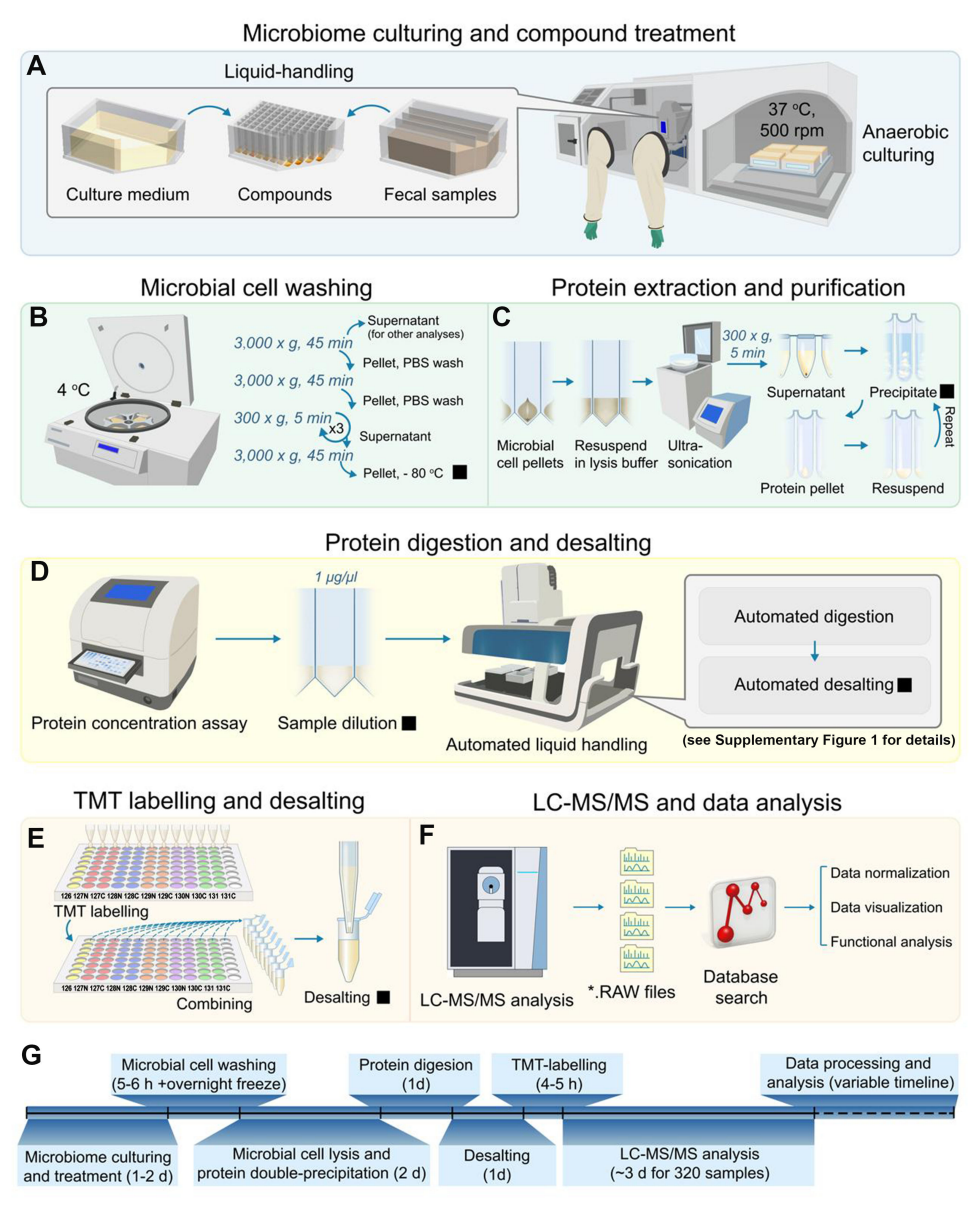
Overview of the RapidAIM 2.0 protocol workflow. (A) Using an anaerobic chamber with a 37 °C incubator, individual fecal samples are cultured in the optimized medium with or without the stimuli of interest. A 96-well liquid handler is recommended for liquid handling. Sample plates are shaken on an orbital shaker at 500 rpm; (B) Cultured microbiome samples are then washed with PBS buffer using a centrifuge with a deepwell plate rotor; (C) Microbial cells are lysed in 96-well PCR plates using a cup-horn ultra-sonicator, and proteins are then purified using a double-precipitation procedure; (D) Proteins are then quantified and diluted, followed by automated digestion and desalting; (E) Desalted peptides are then labeled with TMT and each TMT11plex^TM^ mix is then desalted again; (F) Samples are finally analyzed with LC-MS/MS, and *.RAW files are subjected to database search and data analysis; (G) Estimated timeline corresponding to an experiment of four 96-well plates (i.e., 320 samples). Filled squares indicate pause points to which samples can be stored at -20 °C until further processed (or otherwise -80 °C if stated). TMT: Tandem mass tags; LC-MS/MS: liquid chromatography - tandem mass spectrometry.

As illustrated in Figure 1, the RapidAIM 2.0 protocol is divided into six sequential stages: (A) microbiome collection, culturing, and compound treatment; (B) microbial cell washing; (C) protein extraction and purification; (D) protein digestion and desalting; (E) TMT labeling and desalting; (F) LC-MS/MS analysis and metaproteomic data analysis. First, individual fecal samples are cultured with or without compounds/stimuli of interest [Figure 1A]. An anaerobic chamber with a 37 °C incubator capable of accommodating an orbital shaker is used. A 96-well liquid handler is recommended for liquid handling. Sample plates are shaken on an orbital shaker at 500 rpm for 18-48 h. Either fresh human fecal samples or -80 °C stored biobank samples can be used for the culturing step. We previously showed that our culturing protocol maintains the functionality of individual microbiomes with both sample types^[18,35]^.

Supplementary Methods 1 and 2 provide details for collecting and processing fresh stool samples, and collecting, processing and biobanking of the samples, respectively.

Next, microbial cells are purified using differential centrifugation and then stored at -80 °C [Figure 1B]. Proteins are then extracted from the cells and purified using a double-precipitation procedure [Figure 1C]. After protein quantification, samples are diluted to a recommended protein concentration of 1 µg/µL, followed by automated digestion and desalting procedures [Figure 1D and Supplementary Figure 1A and B]. For labs without automation capacity, 96-well plate-based manual digestion and desalting procedures can also be used [Supplementary Method 3]. However, we validated that the automation increased reproducibility and sensitivity compared to manual liquid handling [Supplementary Figure 2]. Samples are then labeled using TMT11plex^TM^ reagents pre-aliquoted and dried into 96-well plates [Figure 1E]. TMT labeling is quenched, pooled by rows, and desalted prior to sample analysis using an LC-MS/MS. *.RAW files are subjected to database search using MetaLab software, and subsequent data analysis is preformed [Figure 1F]. A timeline breakdown for the whole protocol is given in Figure 1G.

### Proof-of-concept of the RapidAIM 2.0 approach

**Figure 2:**
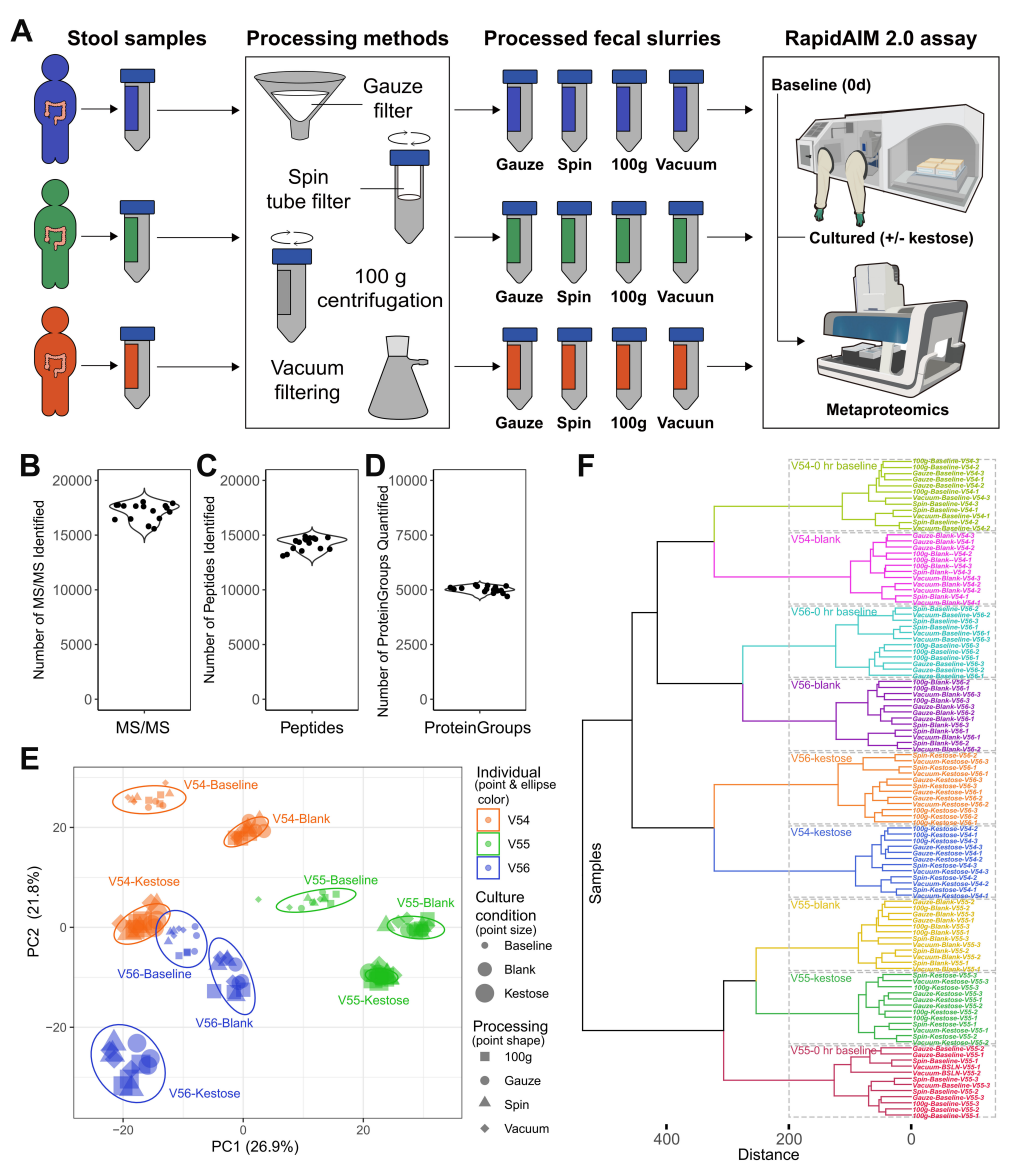
Evaluating the effects of stool sample processing methods. (A) Experimental design. Gauze filtration (Gauze), 100 µm vacuum filtration (Vaccum), 100 µm spin tube filtration (Spin) and 100 *g* spinning (100 *g*) workflows were compared; (B) Number of MS/MS identified in each multiplexed sample set; (C) Number of peptides identified in each multiplexed sample set; (D) Number of protein groups quantified in each multiplexed sample set; (E) Principal component analysis of the sample processing study. Different colors indicate fecal samples from three different individuals. Sizes of data points represent different conditions (small - 0 h baseline, medium - blank, large - kestose). Four different shapes indicate different processing methods. Ellipses indicate the 95% confidence interval for each individual-condition subgroup; (F) Hierarchical clustering of individual samples processed using different sample processing protocols and treated with or without kestose.

Here, we exemplify the described RapidAIM 2.0 workflow with a demonstration study, in which we evaluated the use of different human stool sample collection and biobanking processing workflows on individual gut microbiome responses to kestose, a prebiotic oligosaccharide as of established knowledge of its impact on gut metaproteomes^[34]^ [Figure 2A]. All processed fecal samples were stored at -80 °C before performing the RapidAIM 2.0 experiment. Samples of all comparisons were randomized before being labeled using TMT11plex^TM^. An Ultimate 3000 RSLCnano system coupled to an Orbitrap Exploris 480 was used for the analysis of TMT-labeled samples using a two-hour gradient. Details of LC-MS/MS parameters are as described in Equipment setup. LC-MS/MS *.RAW files were searched against the IGC database^[37]^ using MetaLab 2.3.0.

Altogether, 162 samples were labeled with a TMT11plex^TM^ kit, resulting in 17 multiplexed samples. On average, 17,078 ± 24 MS/MS spectra were identified (MS/MS identification rate 24.2% ± 1.0%), resulting in 14,026 ± 570 identified peptides and 5,014 ± 142 protein groups per multiplexed sample set (Mean ± SD, *N* = 17; Figure 2B-D; Supplementary Figure 3). This is highly comparable to the previous label-free quantification in RapidAIM^[18]^. A substantial proportion (97.3%) of identified peptides belong to bacteria, with the other 2.7% assigned to Eukaryote, specifically human proteins [Supplementary Figure 4]. Dataset was then preprocessed using MSstatsTMT which includes a pipeline of spectrum-level normalization, protein summarization, and protein-level normalization using the LogSum method^[38]^. Multivariate statistical analysis can then be performed using the processed dataset.

We first compared four different sample processing methods on microbiome responses (see Methods). Among whese, the Gauze method has the merit of low cost, the Vacuum filtration method has the merit of rapid processing of large volumes, the Spin method has the merit of easy handling, and the 100 *g* method has the merit of both low cost and easy handling. Principal component analyses (PCA) show that all technical triplicates were well-clustered [Figure 2E]. PCA and hierarchical clustering [Figure 2E and F] also show that for all three tested individual microbiomes, samples were clustered by individual and condition (0 day baseline, blank, and kestose). No separation between the four different stool sample processing strategies was observed, suggesting that all four strategies were applicable for RapidAIM without impact on microbiome functional responses and that one strategy can be selected based on the evaluation of cost and time preferences.

**Figure 3:**
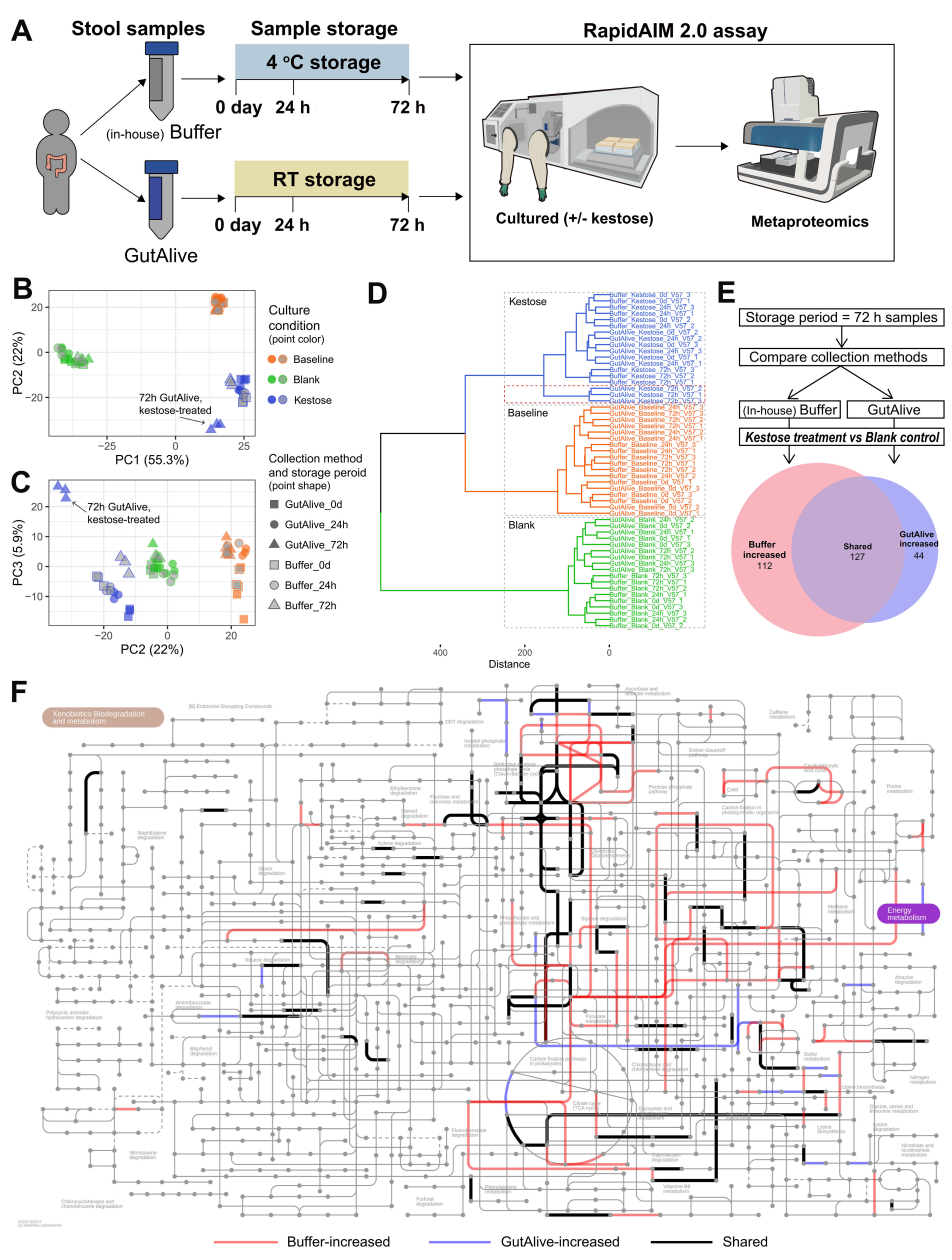
Comparison of sample storage condition and time on pathway responses. (A) Samples collected with the GutAlive kit and stored under room temperature for 24 and 72 h were compared with samples collected with our in-house buffer and stored at 4 °C for the same period of time for their maintenance of microbiome functionality by assessing functional responses to kestose using the RapidAIM protocol; (B) PC1 *vs*. PC2; (C) PC2 *vs*. PC3; (D) Hierarchical clustering of samples showing 72-hour samples of GutAlive differentiated from 72-hour samples of our in-house buffer; (E) Comparison of the responses of 72-hour samples to kestose between the (in-house) Buffer group and GutAlive group, Euler plot (area proportional Venn plot) showing numbers of significantly increased COGs by *t*-test (*P* values-adjusted by FDR); (F) Pathways corresponding to significantly increased COGs in response to kestose. COGs only in the GutAlive group (blue lines), only in the Buffer group (red lines), and shared responses (black lines) are shown.

We next assessed the feasibility of preserving samples in specific collection buffers and storing them before undergoing biobanking processing. We tested the storage efficacies of our in-house PBS-glycerol buffer and the commercial GutAlive buffer by comparing samples processed immediately after collection (0 day) with those stored for 24 and 72 h. While the sample collected using the in-house buffer were stored at 4 °C, the GutAlive samples were stored at room temperature [Figure 3A]. Similar to the previous test on sample processing method, the sample storage test showed clear separations by storage conditions. While samples stored for 24 h under room temperature in the GutAlive kit showed no separation from the samples that were processed on the day of collection, the 72-hour sample showed a separation of the metaproteomics profiles from the other groups, indicating a possible change in microbiome functionality that is specific to kestose uptake during the storage [Figure 3B-D]. PERMANOVA analysis showed that the storage period has a more significant impact in the GutAlive than in the in-house buffer group [Supplementary Tables 5 and 6]. Therefore, we performed differential protein abundance analyses between kestose- and blank control- group samples of each preservation buffer, and we annotated proteins with COG and examined pathway responses. We observed that the different preservation methods could influence different numbers of COGs that exhibited a significant increase in the presence of kestose [Figure 3E], and the in-house buffer is sensitive in observing more responded COGs. Next, we mapped the responded COGs to microbial metabolism pathway maps in iPATH and found that in-house buffer storage method can better capture microbial metabolic pathway responses [Figure 3F]. The result suggests that storage of samples in our in-house buffer at 4 °C for 72 h prior to culturing/biobanking does not affect functional responses.

## DISCUSSION

There have been various *in vitro* models to evaluate microbiome responses. Early *in vitro* gut microbiome models were based on large-scale bioreactors that are low-throughput and, due to the large volume of cultures, very costly owing to the considerable amount of compounds added. More recent advances in modeling the gut ecosystem include realizing the culturing of complex human gut microbiome in anaerobic intestine-on-a-chip models, enabling the observation of host-microbiome interactions^[10]^. However, for the purpose of high-throughput compound screening, these models are not easily adaptable. This study describes the most recently optimized 2.0 version of RapidAIM, which consists of extensive details on stool sample collection, biobanking, *in vitro* culturing and stimulation, microbiome sample processing, metaproteomics measurement and data analysis. Using RapidAIM 2.0, we show consistent responses of individual microbiomes to prebiotic kestose across five different biobanking workflows; we also show that kestose had consistent functional effects across individuals and can be used as a positive control in the assay.

In addition to the recommendations described in the protocol in the Method section, we recommend the following considerations for experimental design:

(1) Plate layout. The experimental design will be performed based on a 96-well format. Taking into consideration the use of TMT11plex^TM^, we recommend that an 8 rows × 10 columns plate layout is used for each 96-well plate. The first column will later be used for the TMT reference sample, which will be generated after the desalting step. The last column will be left blank throughout the experiment.

(2) Randomization. Compound treatments across all assay plates should be randomized. We provide the “96-well plate randomizer” tool in our iMetaLab Suite^[39]^ to assist researchers with the study randomization (https://shiny.imetalab.ca/96_well_randomizer/). Randomizing within and across sample plates will be helpful to detect batch effects between plates, if any, and meet the criteria to apply batch removal tools^[40]^. Samples should be randomized again prior to LC-MS/MS analysis.

(3) Controls. Vehicle controls, which are microbiomes cultured in the absence of compound treatment but in the presence of compound vehicle, should be included. For example, when compounds are pre-dissolved in DMSO, the same amount of DMSO should be used in the vehicle control. Positive controls of known effects on the *ex vivo* human gut microbiome should also be included, such as fructooligosaccharide (FOS) or kestose. In addition, a blank control of culture media without inoculation of microbiome should be added to monitor potential contamination occurrences.

(4) Biological and technical replications. An adequate number of biological replicates should be included and the sample size should be determined through power analysis^[41]^. In terms of technical replicates, for smaller-scale studies or individualized studies, we recommend that each condition is carried out with technical triplicates. For large-scale studies that have sufficient power of biological replicates, the minimum requirement is that technical triplicates of negative and positive controls should be performed.

(5) Quality controls for LC-MS/MS. For large-scale assays requiring a relatively long LC-MS/MS running time (e.g. > 2 days), quality control (QC) samples are necessary to ensure quality, reproducibility, and comparability of results across different batches. The importance of conducting QC runs also includes ensuring the cleanliness of the samples. We strongly suggest that the QC sample should be study-specific. For this, a mixture of a small aliquot from each sample is recommended. It is also recommended that the researchers run the QC samples on LC-MS/MS to confirm sample quality before labeling by TMT.

(6) Enabling more analysis. As metaproteomics provides information on protein compositions, it does not include other information such as genomic and metabolite compositions. We recommend that to enable multi-omics analysis and other biochemical analysis of the samples, aliquots of samples should be saved at certain steps for such purposes. For example, the microbial cell-free supernatant of the microbial cell washing step may be taken/stored for possible metabolite analysis. At the final step of microbial cell washing, before pelleting the microbial cells, samples may be divided into two aliquots for metaproteomics and metagenomics, respectively. In addition, this protocol was developed for the aim of compound screening purpose which does not directly enable deep metaproteomics analysis. We recommend that the researchers save aliquots of protein lysate or digest for each sample and do fractionation-based deep metaproteomics with specific samples of interest following the first-pass screening analysis.

## DECLARATIONS

### Availability of data and materials

Source codes for the results section are provided as an RNotebook file, accessible at https://github.com/northomics/RapidAIM_protocol_dataset.

### Copyright

© The Author(s) 2024.
